# COVID-19 in immunocompromised patients after hematopoietic stem cell transplantation: a pilot study

**DOI:** 10.1097/BS9.0000000000000183

**Published:** 2024-01-25

**Authors:** Zilu Zhang, Jingtao Huang, Luxiang Wang, Zengkai Pan, Jiayu Huang, Chuanhe Jiang, Sujiang Zhang, Su Li, Xiaoxia Hu

**Affiliations:** a National Research Center for Translational Medicine, State Key Laboratory of Medical Genomics, Shanghai Institute of Hematology, Ruijin Hospital, Shanghai JiaoTong University School of Medicine, Shanghai 200025, China; b GoBroad Medical Institute of Hematology (Shanghai Center), Shanghai 201418, China; c Collaborative Innovation Center of Hematology, Shanghai JiaoTong University School of Medicine; Shanghai 200025, China

**Keywords:** Hematopoietic stem cell transplantation, Immune reconstitution, Immunocompromised patients, Severe acute respiratory syndrome coronavirus 2

## Abstract

Data on severe acute respiratory syndrome coronavirus 2 (SARS-CoV-2) infection in patients at early stage of immune reconstitution after hematopoietic stem cell transplantation (HSCT) are limited. In the present study, we retrospectively investigated the incidence and clinical features of SARS-CoV-2 infection in patients who underwent HSCT in 2022. Patients (allo-HSCT, n = 80; auto-HSCT, n = 37) were consecutively included in the study. The SARS-CoV-2 infection rate was 59.8%, and the median interval of HSCT to coronavirus disease 2019 (COVID-19) was 4.8 (range: 0.5–12) months. Most patients were categorized as mild (41.4%) or moderate (38.6%), and 20% as severe/critical. No deaths were attributable to COVID-19. Further analysis showed that lower circulating CD8^+^ T-cell counts and calcineurin inhibitor administration increased the risk of SARS-CoV-2 infection. Exposure to rituximab significantly increased the probability of severe or critical COVID-19 compared with that of mild/moderate illness (*P* < .001). In the multivariate analysis, rituximab use was associated with severe COVID-19. Additionally, COVID-19 had no significant effect on immune reconstitution. Furthermore, it was found that Epstein–Barr virus infection and rituximab administration possibly increase the risk of developing severe illness. Our study provides preliminary insights into the effect of SARS-CoV-2 on immune reconstitution and the outcomes of allo-HSCT recipients.

## 1. INTRODUCTION

The global coronavirus disease 2019 (COVID-19) pandemic that originated due to infection with severe acute respiratory syndrome coronavirus 2 (SARS-CoV-2) continues to pose daunting challenges, especially to vulnerable individuals, including cancer survivors. SARS-CoV-2 causes acute respiratory infections followed by hyperinflammation in a subset of patients who develop severe disease. As of 2023, the global death toll exceeded six million.^1^ A comprehensive meta-analysis indicated notably higher mortality rates in patients with hematological malignancies than in those with solid tumors (odds ratio = 1.64).^2,3^ Similarly, immune deficiency is expected to have a prolonged effect on the progression and outcome of SARS-CoV-2 infection.^4^ In a prospective cohort of patients with aplastic anemia, the percentage of patients with severe/critical COVID-19 was similar to that of the general population (0.6% vs 0.5%, respectively, *P* > .05)^5^ but was substantially lower than that of patients with hematological malignancies.^6^ However, uncertainty surrounds the outcomes of SARS-CoV-2 infection in transplant recipients with underlying hematologic malignancies. These individuals are characterized by profound immune dysregulation caused by conditioning, acute/chronic graft-vs-host disease (GvHD), and long-term immunosuppressive therapy,^7^ rendering them particularly susceptible to SARS-CoV-2 infection. Therefore, the Worldwide Network for Blood and Marrow Transplantation and the Center for International Blood and Marrow Transplant Research have issued guidelines to protect hematopoietic stem cell transplantation (HSCT) recipients and donors during the COVID-19 pandemic.^8^ However, their susceptibility to infection persists^9,10^ and adequate data on the outcomes of transplant recipients with COVID-19 are scarce.

In December 2022, the worldwide spread of SARS-CoV-2 became notable, exposing all HSCT recipients to infection risk. The first year after transplantation is characterized by cellular and humoral immunodeficiencies and greater susceptibility to infection. In the present study, we aimed to characterize the clinical characteristics and outcomes of COVID-19 in HSCT recipients within the first year after HSCT during the first surge of the pandemic. By evaluating infections and outcomes after SARS-CoV-2 exposure, our study provides preliminary evidence for optimizing the prevention and control of potential SARS-CoV-2 infections among transplant recipients in the future.

## 2. MATERIALS AND METHODS

### 2.1. Patients

This retrospective study was based on the transplant databases of Shanghai Ruijin Hospital and Shanghai Liquan Hospital. Consecutive hematologic patients receiving HSCT from January 1, 2022, to December 31, 2022, were screened. The eligibility criteria were as follows: age ≥ 16 years and available results of molecular diagnostic tests (real-time polymerase chain reaction) and/or antigen detection of SARS-CoV-2. The exclusion criteria were as follows: incomplete medical information and lack of follow-up data. The final follow-up was conducted on January 31, 2023. The study was approved by the institutional review board of each participating hospital (approval number: 2021-173) and conducted in accordance with the Declaration of Helsinki.

### 2.2. Transplant regimens, GvHD prophylaxis, and infection prophylaxis

The protocols for the preconditioning regimen, GvHD prophylaxis and treatment, and infection prophylaxis have been reported in detail.^11,12^

### 2.3. Definition

SARS-CoV-2 infection was diagnosed using molecular diagnostic tests and/or antigen detection. COVID-19 severity was classified according to the international recommendations of the National Institutes of Health: asymptomatic or pre-symptomatic infection (positive for SARS-CoV-2 virology test without clinical symptoms), mild (upper respiratory symptoms), moderate (acute lower respiratory tract infection, but no hypoxemia), severe (hypoxia ≤ 93% without supplemental oxygen), or critical (admission to intensive care unit). Patients were followed up for a minimum of 30 days after SARS-CoV-2 infection. Cytomegalovirus (CMV) viremia and Epstein–Barr virus (EBV) viremia were detected in the plasma samples at any level of CMV/EBV DNA. CMV disease was diagnosed according to the established criteria.^13^ Mixed infection was defined as the co-occurrence of SARS-CoV-2 and other pathogens in the same infection episode and/or different infection sites involved in the same episode.

### 2.4. Immune recovery

Immune recovery was monitored pre–SARS-CoV-2 infection (15–30 days before SARS-CoV-2 exposure) and after infection (30–45 days after SARS-CoV-2 exposure) using flow cytometry. Peripheral blood mononuclear cells were stained with fluorochrome-labeled monoclonal antibodies against cluster of differentiation (CD) cell surface molecules. We determined the absolute numbers of 13 lymphocyte subpopulations via immunophenotyping: CD3^+^ T, CD4^+^ T, CD8^+^ T, CD4^+^CD25^+^ regulatory T, CD4^+^CD45RA^+^ naive T, CD4^+^CD45RO^+^ memory T, CD4^+^CD45^+^CD127^low^ regulatory T, CD3^+^HLA-DR^+^ activated T, CD3^+^CD69^+^ early activated T, CD4^+^CD28^+^ functional T, CD8^+^CD28^+^ functional T, CD19^+^ B, and CD56^+^ NK cells.

### 2.5. Data acquirement

The investigators at each hospital used the institutional electronic medical records of clinical databases to obtain the required information. Collected data included patient demographics, diagnoses, transplant regimens, and clinical outcomes (relapse, mortality, and survival). The clinical data were collected using an electronic questionnaire. All data were independently reviewed by 2 experienced transplantation physicians to ensure the accuracy of the results.

### 2.6. Statistical analysis

Frequencies and percentages were used to describe patient characteristics. Univariate and multivariate analyses of the clinical, laboratory, and therapeutic variables associated with outcomes were performed using logistic regression models. For multivariate analysis, variables with parameter estimates with *P* ≤ .10 in the univariate analysis were included. Univariate and multivariate Cox regression analyses were performed to determine the effect of potential prognostic factors on the clinical outcomes. Two-sided exact *P* values were reported and *P* ≤ .05 was considered statistically significant. Statistical analyses were performed using Statistical Package for the Social Sciences version 26 (SPSS Inc.; IBM, Armonk, New York).

## 3. RESULTS

### 3.1. Acquisition, incubation, and shedding

**Figure 1.**
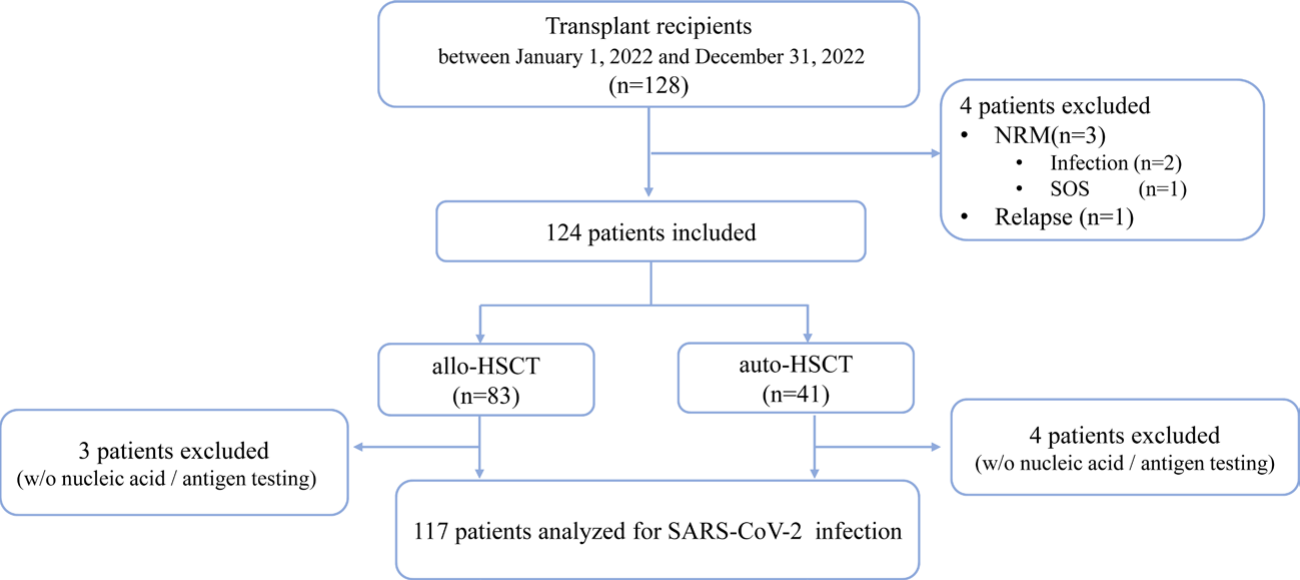
Flowchart of the study. allo-HSCT = allogeneic hematopoietic cell transplantation, auto-HSCT = autologous hematopoietic cell transplantation, NRM = non-relapse mortality, SARS-CoV-2 = severe acute respiratory syndrome coronavirus 2, SOS = sinusoidal obstruction syndrome.

Between January 1, 2022, and December 31, 2022, 128 patients consecutively received HSCT. Three patients died of non-relapse mortality and 1 patient experienced early relapse 60 days after HSCT. A total of 124 patients were enrolled for further analyses. We excluded 7 patients who did not undergo nucleic acid/antigen testing. SARS-CoV-2 infection was confirmed in 70 patients, and the remaining 47 uninfected patients were included in the follow-up study. The median follow-up duration was 103 days (range: 77–138 days). Eighty-three patients received allogeneic HSCT (allo-HSCT) and 41 received autologous HSCT (auto-HSCT) (**Fig. 1**). The incidences of SARS-CoV-2 infection were 44% and 70.3% in the allo-HSCT and auto-HSCT groups, respectively. The duration of infection was significantly longer in the allo-HSCT than in the auto-HSCT group (Supplemental Table 1, http://links.lww.com/BS/A84). The median interval from transplantation to SARS-CoV-2 exposure was equivalently distributed from 1 to 12 months. The SARS-CoV-2 infection rates at 1 to 3, 4 to 6, 7 to 9, and 10 to 12 months after HSCT were 55.5%, 55.8%, 66.6%, and 55.9%, respectively (Supplemental Figure 1, http://links.lww.com/BS/A84).

### 3.2. Clinical characteristics of patients with SARS-CoV-2 infection

The patient characteristics at the time of COVID-19 diagnosis are summarized in Table 1. The median time from transplantation to SARS-CoV-2 infection was 4.8 months (range: 0.5–12 months). Nucleic acid tests were used to diagnose 51.4% (36/70) of infections. At the time of COVID-19 diagnosis, 29 (41.4%) patients were treated with calcineurin inhibitors (CNIs), and 5 (7%) were treated with tyrosine kinase inhibitors (TKIs). The most common symptoms of COVID-19 were fever (77.1%; n = 54), cough (71.4%; n = 50), and expectoration (52.9%; n = 37). When stratified by disease severity, 41.4% (29/70) of the patients had mild, 38.6% (27/70) of the patients had moderate, and 20% (14/70) of the patients had severe/critical disease.

**Table 1:** Patient characteristics at diagnosis of COVID-19.

Clinical characteristics	SARS-CoV-2 infection (n = 70)
Interval from HSCT to COVID-19, month (range)	4.8 (0.5–12.5)
Method of diagnosis, n (%)	
Nucleic acid	36 (51.4)
Antigen	34 (48.6)
Vaccination, n (%)	58 (82.9)
Severity of COVID-19, n (%)	
Mild	29 (41.4)
Moderate	27 (38.6)
Severe/critical	14 (20)
Interventions, n (%)	
Hospitalization	21 (30)
Corticosteroid therapy	14 (20)
Oxygen support	32 (45.7)
Antiviral therapy	33 (47.1)
Abnormal radiological pulmonary finding, n (%)	28 (40)
Mixed infection, n (%)	32 (45.7)
Bacterial	32 (45.7)
Viral except COVID-19	4 (5.7)
fungal	19 (27.1)
Duration of COVID-19 infection, n (%)	
≤10 d	32 (45.7)
11–20 d	18 (25.7)
21–40 d	9 (12.9)
NA	11 (15.7)
Outcome, n (%)	
Recovery	68 (97.1)
Progressed	2 (2.9)

COVID-19 = coronavirus disease 2019, HSCT = hematopoietic stem cell transplantation, NA = not available, SARS-CoV-2 = severe acute respiratory syndrome coronavirus 2.

**Figure 2.**
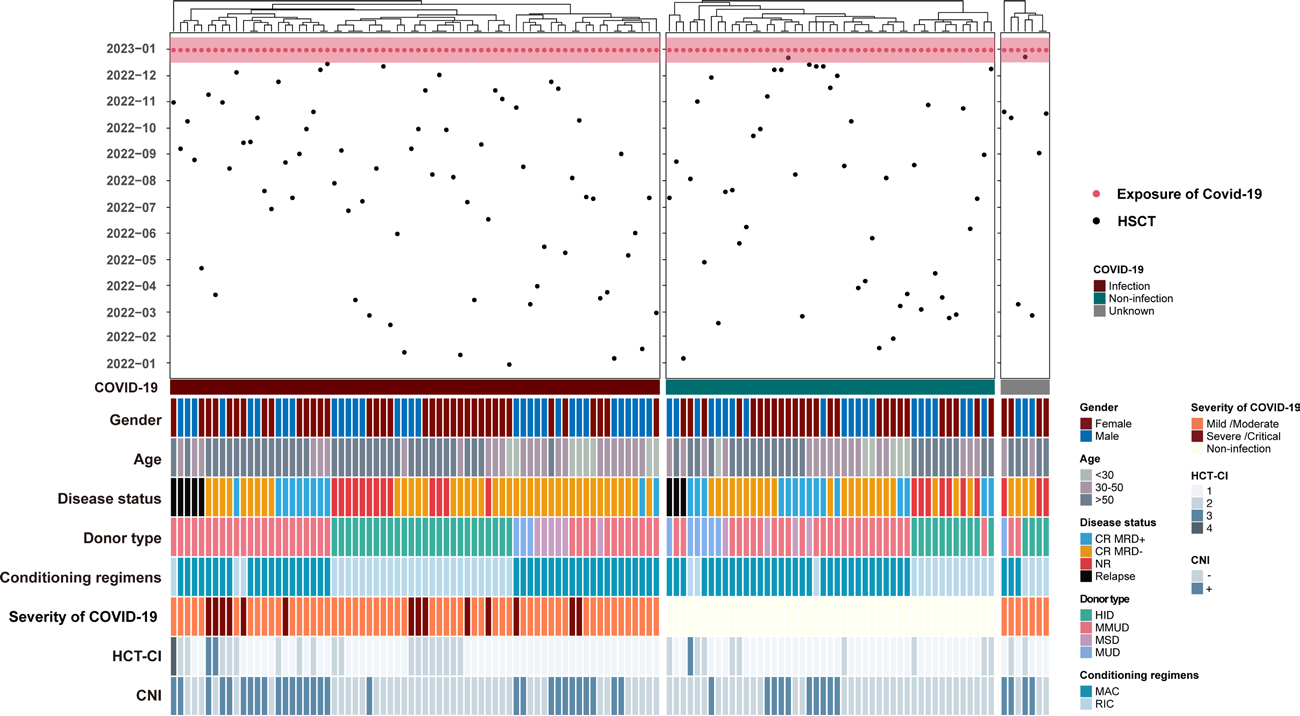
Swimmer plot of clinical characteristics among HSCT patients at diagnosis of COVID-19. CNI = calcineurin inhibitor, COVID-19 = coronavirus disease 2019, CR = complete remission, HCT-CI = hematopoietic cell transplantation-comorbidity index, HID = haploidentical donor, HSCT = hematopoietic cell transplantation, MAC = myeloablative conditioning, MMUD = mismatched unrelated donor, MRD = measurable residual disease, MSD = matched sibling donor, MUD = matched unrelated donor, NR = non remission, RIC = reduced intensity conditioning.

We compared the clinical characteristics of patients with and without SARS-CoV-2 infection (**Fig. 2**, Supplemental Table 2, http://links.lww.com/BS/A84). No significant differences in baseline characteristics were observed. A higher proportion of patients with SARS-CoV-2 were with CNIs maintenance compared with their counterparts (41.4% vs 21.3%, respectively, *P* = .028). Twenty-one patients (30%) were hospitalized for COVID-19, and 2 (2.9%) required intensive care. Most patients (54.3%, n = 38) did not require oxygen supplementation, and the remaining 45.7% required a nasal cannula or high-flow oxygen supplementation. The duration of SARS-CoV-2 infection varied, with a median of 9 days (range: 5–40 days). A total number of 68 (97.1%) patients fully recovered from COVID-19 infection, and the other 2 patients progressed to septic shock.

Fifty-four patients underwent chest computed tomography (CT), and 18 (33.3%) were diagnosed with COVID-19–related pneumonia. Four patients manifested bilateral ground-glass changes, and 14 patients had bilateral consolidation (Supplemental Figure 2, http://links.lww.com/BS/A84). Of the 18 patients, 14 (77.8%) had peripherally distributed lesions, 13 (72.2%) had involvement of the basal segment of the lower lobe, and 4 (22.2%) had ground-glass opacities. Three suspected patients tested negative for SARS-CoV-2 via nucleic acid testing with a nasopharyngeal swab but were diagnosed using bronchoalveolar lavage fluid (BALF) with next-generation metagenomic sequencing testing.

### 3.3. Mixed infection in patients with SARS-CoV-2 infection

Mixed infections were diagnosed in 32 patients (45.7%), with bacteria being the most common pathogen (n = 32, 45.7%) followed by fungi (n = 19, 27.1%). Patients in the mixed infection group were more likely to develop severe or critical disease than patients in the non-mixed infection group (43.9% vs 0%, respectively, *P <* .001). Compared with that in the severe COVID-19 patients without mixed infection, the duration of infection was markedly longer in the mixed infection group (13 days [range: 7–40 days] vs 9 days [5–37 days], respectively, *P* = .03). Moreover, CMV disease, EBV reactivation, and rituximab use were more commonly observed in the mixed infection group (Table 2).

**Table 2:** The comparison of coinfection and non-coinfections of SARS-CoV-2.

Clinical characteristics	Mixed infection (n = 32)	Non-mixed infections (n = 38)	*P* value
Sex, n (%)			.092
Male	19 (59.4)	14 (36.8)	
Female	13 (40.6)	24 (63.2)	
Age, y (range)	48 (16–74)	47 (16–67)	
Severity of COVID-19, n (%)			<.001
Mild	1 (3.1)	28 (73.7)	
Moderate	17 (53.2)	10 (26.3)	
Severe/critical	14 (43.7)	0	
HCT-CI score, n (%)			.46
0	22 (68.8)	27 (71.1)	
1–2	8 (25)	10 (26.3)	
3–4	2 (6.2)	0	
≥5	0	1 (2.6)	
Donor type, n (%)			.217
HID	14 (43.8)	21 (55.3)	
MSD	1 (3.1)	5 (13.2)	
URD	2 (6.2)	1 (2.6)	
Autologous	15 (6.9)	11 (28.9)	
Conditioning regimens, n (%)			.227
MAC	16 (50)	25 (65.8)	
RIC	16 (50)	13 (34.2)	
ATG dosage in HSCT, n (%)			.631
≥5 mg/kg	16 (50)	16 (42.1)	
0–5 mg/kg	16 (50)	22 (57.9)	
Vaccination, n (%)			.601
0	9 (28.1)	7 (18.4)	
1 dose	3 (9.4)	2 (5.3)	
2 doses	14 (43.8)	17 (44.7)	
3 doses	6 (18.7)	12 (31.6)	
Acute GvHD grade II–IV before COVID-19, n (%)			.719
No	29 (90.6)	33 (86.8)	
Yes	3 (9.4)	5 (13.2)	
Chronic GvHD before COVID-19, n (%)			1
No	30 (93.8)	36 (94.7)	
Yes	2 (6.2)	2 (5.3)	
CMV viremia, n (%)	13 (40.6)	14 (36.8)	.808
CMV disease, n (%)	4 (12.5)	0	.039
EBV viremia, n (%)	13 (40.6)	6 (15.8)	.03
Administration of rituximab before COVID-19, n (%)	9 (28.1)	2 (5.2)	.018
Administration of immunosuppressive agents before COVID-19			
CNI, n (%)	11 (34.3)	18 (47.4)	.334
TKI, n (%)	1 (3.1)	4 (10.5)	.366

ATG = anti-thymocyte globulin, CMV = cytomegalovirus, CNI = calcineurin inhibitor, COVID-19 = coronavirus disease 2019, EBV = Epstein–Barr virus, GvHD = graft-vs-host disease, HCT-CI = hematopoietic cell transplantation-comorbidity index, HID = haploidentical donor, MAC = myeloablative conditioning, MSD = matched sibling donor, RIC = reduced intensity conditioning, SARS-CoV-2 = severe acute respiratory syndrome coronavirus 2, TKI = tyrosine kinase inhibitor, URD = unrelated donor.

### 3.4. Comparison of mild/moderate and severe/critical infections

**Figure 3.**
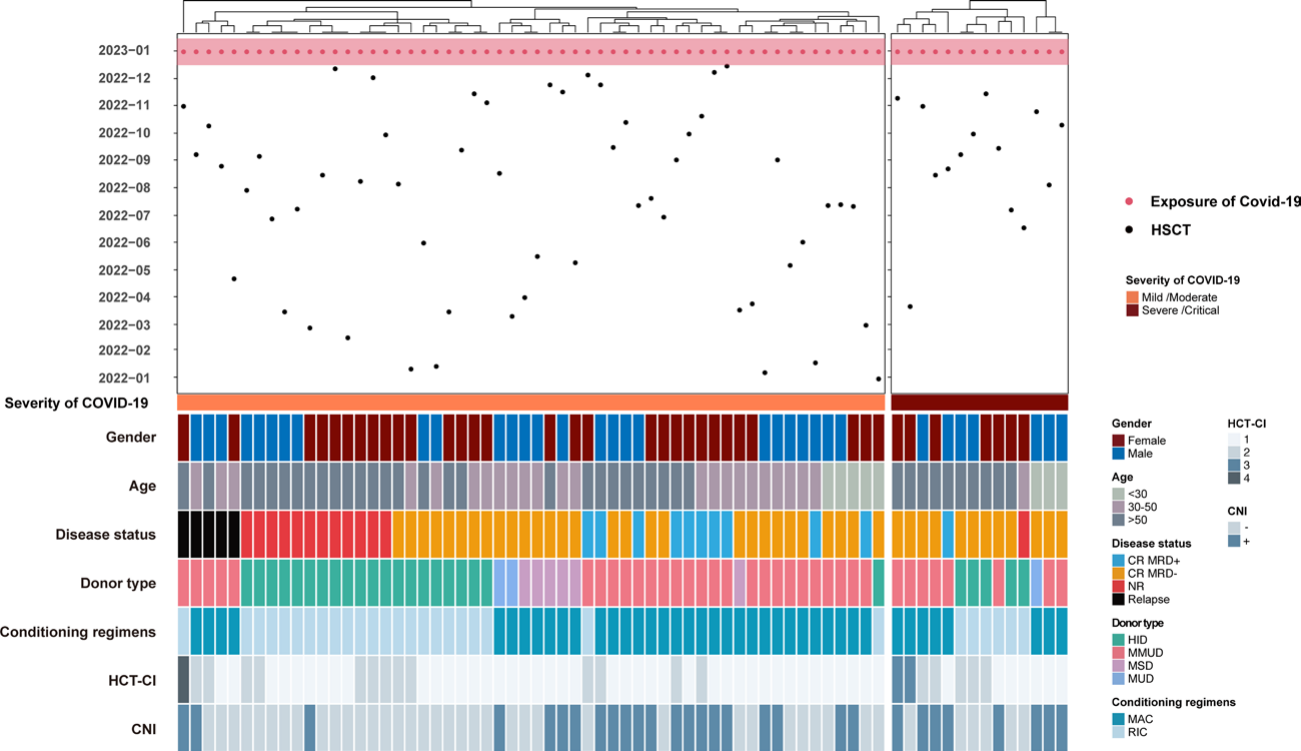
Swimmer plot of clinical characteristics among mild/moderate and severe/critical SARS-CoV-2 infections. CNI = calcineurin inhibitor, COVID-19 = coronavirus disease 2019, CR = complete remission, HCT-CI = hematopoietic cell transplantation-comorbidity index, HID = haploidentical donor, HSCT = hematopoietic cell transplantation, MAC = myeloablative conditioning, MMUD = mismatched unrelated donor, MRD = measurable residual disease, MSD = matched sibling donor, MUD = matched unrelated donor, NR = non remission, RIC = reduced intensity conditioning, SARS-CoV-2 = severe acute respiratory syndrome coronavirus 2.

Next, we compared the clinical characteristics of patients with mild/moderate and severe/critical SARS-CoV-2 infections. Notably, patients in the severe/critical group had higher hematopoietic cell transplantation-comorbidity index (HCT-CI) scores (14.3% vs 1.8%, respectively, *P* = .035), more EBV reactivation episodes (57.1% vs 19.6%, respectively, *P* = .015), and a higher incidence of CMV disease (21.4% vs 1.8%, respectively, *P* = .023) than patients in mild/moderate group. CMV reactivation did not increase the risk of severe or critical illness, whereas CMV disease did (21.4% vs 1.8%, respectively, *P* = .023) (**Fig. 3**, Supplemental Table 3, http://links.lww.com/BS/A84).

**Figure 4.**
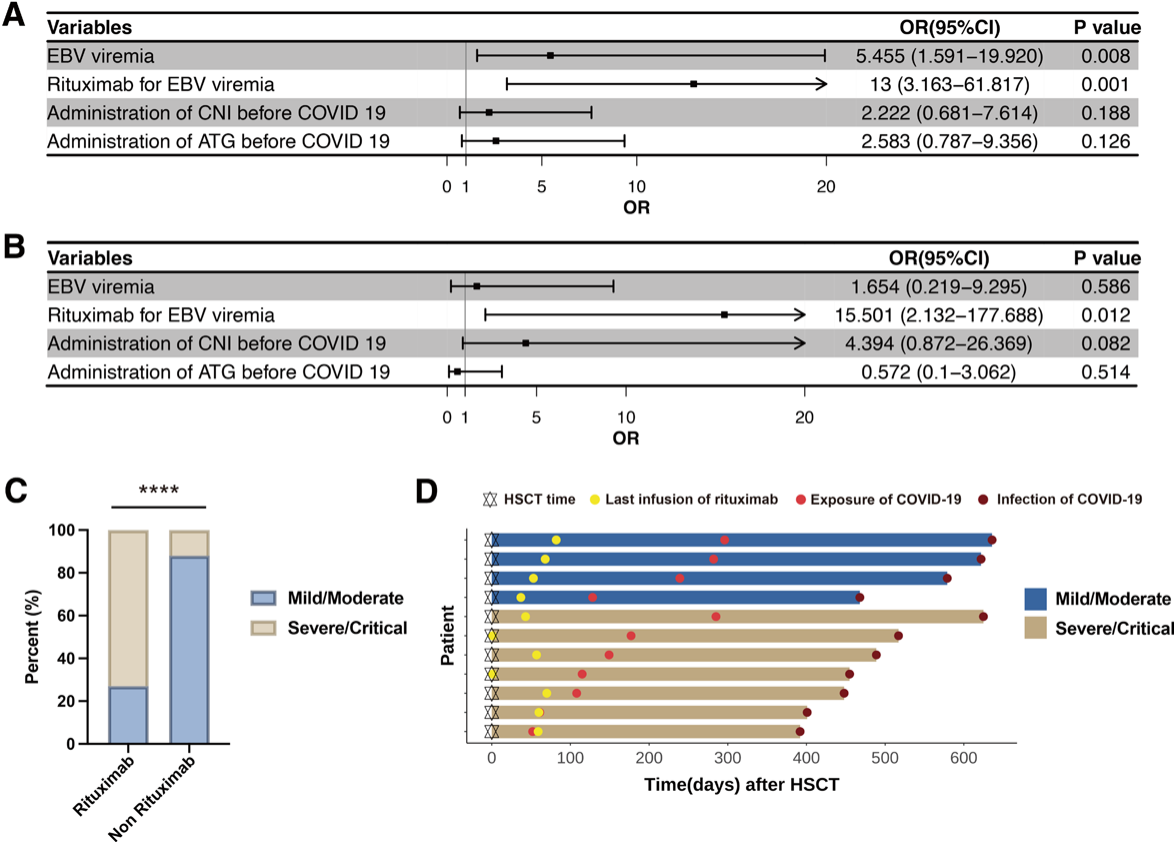
Factors affecting the severity of COVID-19 infection. Univariate (A) and multivariate (B) analysis of severe/critical illness predictors at diagnosis of COVID-19. (C) Comparison of severity between COVID-19 patients with or without rituximab administration. (D) Swimmer plot of the last infusion of rituximab and SARS-CoV-2 infection. ****P < .0001. ATG = anti-thymocyte globulin, CI = confidence interval, CNI = calcineurin inhibitor, COVID-19 = coronavirus disease 2019, EBV = Epstein–Barr virus, HSCT = hematopoietic cell transplantation, OR = odds ratio, SARS-CoV-2 = severe acute respiratory syndrome coronavirus 2.

We conducted univariate and multivariate logistic regression analyses to determine the association between SARS-CoV-2 infection and disease severity. Administration of immunosuppressive drugs (CNIs and/or TKIs) was associated with higher SARS-CoV-2 infection rates (Supplemental Figure 3A and B, http://links.lww.com/BS/A84); a total of 74.36% of patients (29/39) with CNI maintenance were COVID-19 infected, which was notably higher than that in patients without CNI maintenance (52.56%, 41/78, *P* = .028) (Supplemental Figure 3C, http://links.lww.com/BS/A84). Similar results were not observed after anti-thymocyte globulin (ATG) treatment (Supplemental Figure 3D, http://links.lww.com/BS/A84). Additionally, considering the severity of SARS-CoV-2 infection, CNI administration did not increase the risk of severe/critical disease (**Fig. 4A and B**); however, both univariate and multivariate analyses showed that rituximab administration was associated with an increased risk of severe COVID-19 infection (**Fig. 4A and B**). Severe/critical patients were observed in the rituximab group compared to the without rituximab administration group (73.33% vs 11.86%, respectively, *P* < .0001; **Fig. 4C**). The swimmer plot showed the interval between the last rituximab infusion and COVID-19 infection (**Fig. 4D**). The median time from the last rituximab administration to COVID-19 exposure was shorter in severe/critical group than that in mild/moderate-infection group (92 days [range: −7 to 242 days] vs 200 days [range: 91–214 days], respectively, *P* = .145).

### 3.5. Immune reconstitution

Immune reconstitution analysis was conducted in 58 of the 80 allo-HSCT patients. The results showed that lower numbers of CD8^+^ T cells were associated with increased SARS-CoV-2 infection (505/μL vs 1045/μL, respectively, *P* = .033, Table 3). The other lymphocyte subsets did not differ significantly between the infected and non-infected groups. The distribution of lymphocyte subsets before SARS-CoV-2 exposure did not relate to COVID-19 severity.

**Table 3:** Evaluation of lymphocyte subsets.

Lymphocyte subsets counts (cells/μL)	Infection	Without infection	*P* value
Lymphocyte	1.52 (0.2–4.4)	2 (0.21–5.6)	.187
CD3^+^ T cells	697 (2–3765)	1402 (337–4486)	.039
CD4^+^ T cells	167 (1–545)	188 (50–742)	.122
CD8^+^ T cells	505 (1–3359)	1045 (202–4071)	.033
CD56^+^ NK cells	241 (0–1570)	171 (2.2–1024)	.157
CD19^+^ B cells	80 (0–559)	110 (0–475)	.663
CD4^+^CD25^+^ regulatory T cells	1.3 (0.027–16.27)	1.4 (0.23–7.63)	.472
CD4^+^CD45RA^+^ naive T cells	0.66 (0–9.73)	0.795 (0.2–26.8)	.255
CD4^+^CD45RO^+^ memory T cells	16.2 (0.02–51.2)	15.8 (1.89–43)	.744
CD4^+^CD45^+^CD127^low^ regulatory T cells	1.36 (0.02–9.11)	1.3 (0.12–3.6)	.646
CD3^+^HLA-DR^+^ activated T cells	15.3 (0–105.6)	12.5 (3.08–164.9)	.659
CD3^+^CD69^+^ early activated T cells	2.78 (0–21.15)	2.02 (0.27–5.29)	.501
CD4^+^CD28^+^ functional T cells	15.2 (0.22–54.03)	12.19 (2.52–32.25)	.808
CD8^+^CD28^+^ functional T cells	10.43 (0.14–36.02)	5.4 (2.94–75.5)	.921

CD = cluster of differentiation.

**Figure 5.**
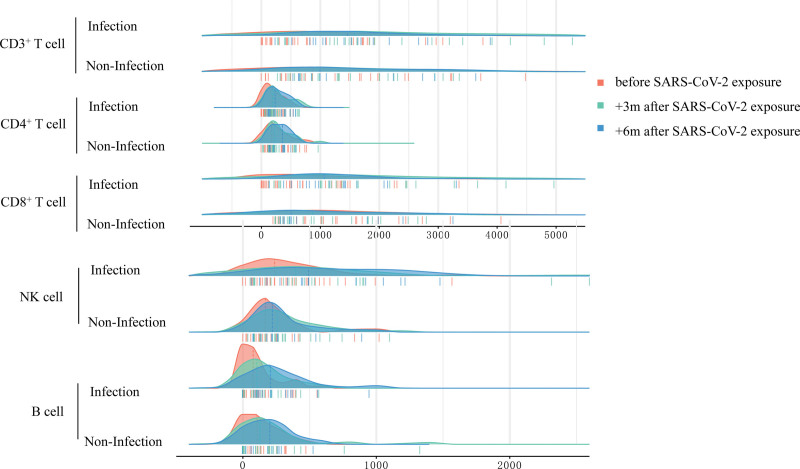
Immune subsets within 6 mo after SARS-CoV-2 exposure. SARS-CoV-2 = severe acute respiratory syndrome coronavirus 2.

Recovery of the immune system after HSCT is highly dynamic. To evaluate the long-term effect of SARS-CoV-2 infection on immune reconstitution, we tracked the lymphocyte subsets of these patients 3 and 6 months after SARS-CoV-2 exposure. There were no differences in the absolute counts of CD3^+^, CD4^+^, CD8+, NK, and B cells between the infected and non-infected groups at any time point. These results indicate that SARS-CoV-2 has a subtle effect on immune reconstitution (**Fig. 5**).

## 4. DISCUSSION

To date, the global number of detected COVID-19 cases is more than 35 million.^14^ Most studies conducted in the pre-vaccine era reported mortality rates higher than 40% among patients with hematological malignancies.^15,16^ However, recent studies have reported an average mortality rate of 4% (1%–20%). The uneven burden of COVID-19 across different populations is a hallmark of this pandemic,^17^ and patients with hematologic malignancies show prolonged viral shedding and higher mortality than the general population and even patients with solid tumors.^18^ Moreover, delayed immunization in patients undergoing HSCT increases the risk of life-threatening sequelae and adverse outcomes of SARS-CoV-2 infection.^19^ New SARS-CoV-2 variants have emerged sequentially, presenting distinct virulence, transmissibility, and host immune responses.^20^ The recently dominant Omicron variants in China are marked by increased transmissibility but decreased lethality.^21^ SARS-CoV-2 Omicron variants have evolved to evade neutralizing antibodies elicited by previous infections and vaccination,^22^ presenting a new challenge for HSCT patients. Therefore, it is imperative to perform a retrospective analysis to understand the recent implications of COVID-19 on patients undergoing HSCT.

In this study, we assessed the incidence and outcomes of COVID-19 in 117 consecutive HSCT recipients. There were no statistically significant differences in age, sex, primary disease, or HSCT characteristics between the infected and non-infected groups, indicating that these factors did not increase the risk of SARS-CoV-2 infection in HSCT recipients. Immunosuppression may increase the risk of SARS-CoV-2 infection, particularly in severe illnesses.^23^ Immunosuppressive agents, including cyclosporine and tacrolimus, were maintained in 31.2% of the patients before SARS-CoV-2 exposure. Although little evidence of immunosuppression as a significant risk factor for SARS-CoV-2 infection and severe disease was observed in a larger cohort of immunosuppressed patients with liver transplants^24^ and inflammatory bowel disease,^25^ our data showed that patients who received CNIs were at an increased risk of SARS-CoV-2 infection (41.4% vs 21.3%, respectively, *P* = .028). The role of TKI as a prominent risk factor for COVID-19 has been established in a cohort of patients with chronic myeloid leukemia cohort.^26^ However, there is no evidence that TKI administration negatively affects outcomes for COVID-19 patients.^27^ In our study, TKIs were used as maintenance therapy for 6 patients after allo-HSCT, 5 of whom developed mild/moderate SARS-CoV-2 infection. However, the influence of TKIs on COVID-19 susceptibility needs to be verified in larger populations.

The clinical profile and overall course of COVID-19 in our study were similar to those in larger cohorts of patients with hematological malignancies who underwent HSCT.^28,29^ Overt symptoms upon initial assessment commonly included fever, cough, and fatigue, a trend evident across all patient groups. Nevertheless, our study found that severe/critical illness accounted for 12% of all infected episodes, which was higher than the proportions reported in other studies on HSCT populations.^28^ This difference could be attributed to the higher proportion of patients who underwent allo-HSCT (68.4%) in our study, which is consistent with previous results of allo-HSCT patients.^30^

Imaging is not routinely indicated for COVID-19 in asymptomatic individuals or patients with mild symptoms unless they are at risk of disease progression. In this study, 54 patients underwent lung CT. Typically, radiologic findings are contingent upon the disease stage,^31^ with frequent radiographic findings in COVID-19 pneumonia, including multifocal consolidation or ground-glass opacity, which may progress to pneumonia.^32^ Of the patients, 33% showed consolidation and/or ground-glass opacities, with no progression to organizing pneumonia. These results confirmed that patients infected with the Omicron variant (B.1.1.529) had a lower risk of pneumonia and clinical severity than those infected with alternative variants, in agreement with previous reports.^33^ Viral load in the lower respiratory tract may persist at a higher level for a prolonged period. Thus, BALF is a more reliable sample for immunocompromised patients compared with nasopharyngeal swab.^34^

More than half the patients in our cohort (n = 49) were monitored at an outpatient clinic. Although several reported risk factors associated with disease severity were noted, they did not show a substantial effect in the present study, likely owing to the small size of our group. None of our patients died of COVID-19 or related complications, which was a considerably better outcome than the outcomes reported previously.^9,35–39^ There are several possible explanations for this discrepancy. First, there have been considerable advances in COVID-19 vaccinations and treatments, including the advent of antiviral medications, which have greatly reduced the incidence of severe or critical infection.^20,40,41^ In addition, Omicron variants exhibit reduced pathogenicity, resulting in weaker inflammatory responses than the other variants.^42^ Cohen et al^43^ reported that the probability of severe illness reduced by 73% during an Omicron-dominated wave.

Most patients undergoing HSCT require scheduled outpatient visits, which could potentially expose them to a variety of pathogens, including SARS-CoV-2. Further exploration of the vulnerability of immunodeficient patients to viral infections remains a priority. The immunological pathogenicity of SARS-CoV-2 is complicated, given its virulence and lack of temporal coordination between innate and adaptive immune responses.^44^ B cell-depleting agents, such as rituximab, which target the CD20 marker on B cells, are widely used for B cell hematological malignancies, EBV reactivation after allo-HSCT, and post-transplant lymphoproliferative disorders.^45^ However, the safety of rituximab in the context of COVID-19 is still under review.^46^ B-cell depletion compromises antiviral immunity, increases the risk of reactivation, and reduces viral clearance. However, it may be beneficial in specific scenarios as a mechanism for regulating adaptive host immune responses in patients with COVID-19. Severe infection and prolonged hospital stay increase the risk of morbidity, mortality, and infection-related sequelae, as observed in a study of patients with inflammatory rheumatic and musculoskeletal diseases treated with rituximab.^47^ In the present study, we identified rituximab as a potential risk factor for severe COVID-19, which has not been reported previously. Additionally, the median interval between COVID-19 exposure and the last rituximab infusion was shorter in severe/critical patients than in those with mild/moderate infection. However, the exact causal relationship needs to be verified in a larger cohort.

Our study is limited by its retrospective design and relatively short follow-up period. Furthermore, cytokines have an important effect on the reconstitution of lymphocyte subsets and immune responses after SARS-CoV-2 infection. Due to the retrospective nature of our study, we could not collect enough data to thoroughly analyze the effect of cytokines on illness. Nonetheless, we believe that our study offers a comprehensive examination specific to HSCT recipients with COVID-19.

In conclusion, our study highlights the outcomes of SARS-CoV-2 infection among HSCT patients during the Omicron variant wave and provides new insights into the risk factors associated with COVID-19 severity. Our data suggest that EBV reactivation and rituximab use would increase the risk of developing severe disease. Ongoing surveillance of immune reconstitution is critical to evaluate the long-term impact of SARS-CoV-2 infection in HSCT recipients.

## AUTHOR CONTRIBUTIONS

X.H., S.L., and S.Z. designed the study; Z.Z. and J.H. wrote the manuscript. Z.Z., J.H., L.W., and Z.P. were involved in collecting, analyzing, or interpreting research data and writing the manuscript. C.J. and J.H. analyzed research data.

## ACKNOWLEDGMENTS

This work was supported by the National Key Research and Development Program of China (no. 2022YFC2502600), the National Natural Science Foundation of China (nos. 82170206, 82300228), and Shanghai Municipal Health Commission Project of Disciplines of Excellence (no. 20234Z0002).

## Supplementary Material
