# Supplementary material for: COVID-19 in immunocompromised patients after hematopoietic stem cell transplantation: a pilot study
Source: Blood Sci. 2024 Jan 25;6(2):e00183. doi: 10.1097/BS9.0000000000000183 (PMC10817160; doi:10.1097/BS9.0000000000000183)
Supplement: Supplementary file 1 [file bs9-6-e00183-s001.pdf]

Contents

Supplementary Tables

Supplemental Table 1: Patient characteristics of SARS-CoV-2 infection

Supplemental Table 2: SARS-CoV-2 infection in HSCT patients

Supplementary Figures

Supplemental Fig.1 Number of HSCT cases and percentage of SARS-CoV-2 infection according to the date of exposure.

Supplemental Fig.2 CT findings of COVID-19 pneumonia (lung window). (A) extensive areas of ground-glass opacity involving bilateral lungs. (B) consolidation with subpleural distribution

Supplemental Fig.3 Factors affecting COVID-19 infection. Univariate (A) and multivariate (B) analysis of predictors at diagnosis of COVID-19. Comparison of COVID-19 infection rate between patients with or without CNI (C) or ATG (D) administration. OR, odds ratio. CI, Confidence Interval. *p<0.05.

Supplemental Tables

Supplemental Table 1: Patient characteristics of SARS-CoV-2 infection

	allo-HSCT (n=80)	auto-HSCT (n=37)	*P* value
**Infection, n (%)**	44(55)	26(70.2)	0.156
**Age, years (range)**	47(16-69)	55(21-74)	0.002
**Mixed infection, n (%)**	17(38.6)	15(57.7)	0.143
**Severity of COVID-19, n (%)**			0.326
Mild	18(40.9)	11(42.3)	
Moderate	17(38.6)	10(38.5)	
Severe /Critical	9(20.5)	5(19.2)	
**Duration of COVID-19 infection, n (%)**			0.004
≤10 d	25(56.8)	7(26.9)	
11-20d	5(11.4)	13(50)	
21-40 d	7(15.9)	2(7.7)	
NA	7(15.9)	4(15.4)	
**Pneumonia, n (%)**	19(43.2)	15(57.7)	0.323
**Oxygen support, n (%)**	27(61.4)	11(42.3)	0.122
**Hospitalization, n (%)**	9(20.5)	10(38.5)	1

Abbreviations: allo-HSCT, allogeneic hematopoietic cell transplantation; auto-HSCT, autologous hematopoietic cell transplantation.NA, not available.

Supplemental Table 2: SARS-CoV-2 infection in HSCT patients

Clinical Characteristics	Infection （n=70）	without-Infection （n=47）	*P* value
**Sex（%）**			0.851
Male	33(47.1)	21(44.7)	
Female	37(52.9)	26(55.3)	
**Age, years (range)**	52(16-74)	51(15-69)	0.628
**Diagnosis, n (%)**			0.496
AML	25(35.7)	23(48.9)	
ALL	11(15.7)	7(14.9)	
MDS	4(5.7)	3(6.4)	
GS	2(2.9)	0	
MM	23(32.9)	12(25.5)	
lymphoma	5(7.1)	1(2.1)	
CMML	0	1(2.1)	
**HCT-CI score, n (%)**			0.87
0	49(70)	37(78.8)	
1-2	18(25.7)	9(19.1)	
3-4	2(2.9)	1(2.1)	
≥5	1(1.4)	0	
**Donor type, n (%)**			0.222
allogenous			
HID	35(50)	26(55.3)	
MSD	6(8.6)	4(8.5)	
URD	3(4.3)	6(12.8)	
autologous	26(37.1)	11(23.4)	
**Graft type, n (%)**			0.436
PB	67(95.7)	43(91.5)	

PB+UCB	3(4.3)	4(8.5)	
**ATG dosage, n (%)**			0.456
≥5mg/kg	32(45.7)	25(53.2)	
0-5mg/kg	38(54.3)	22(46.8)	
**Vaccination, n (%)**			0.879
0	16(25.7)	12(25.5)	
1 dose	4(5.7)	1(2.1)	
2 doses	32(45.7)	21(44.7)	
3 doses	18(25.7)	13(27.7)	
**Conditioning regimens, n (%)**			0.335
MAC	41(58.6)	32(68.1)	
RIC	29(41.4)	15(31.9)	
**Acute GvHD grade II–IV before COVID-19, n (%)**			0.586
No	62(88.6)	40(85.1)	
Yes	8(11.4)	7(14.9)	
**Chronic GvHD before COVID-19, n (%)**			0.712
No	66(94.3)	43(91.5)	
Yes	4(5.7)	4(8.5)	
**Cumulative exposure to steroids**	3.33(0.46-6067)	3.57(0.49-8.33)	0.246
**CMV viremia, n (%)**	27(38.6)	20(42.6)	0.703
**CMV disease, n (%)**	4(5.7)	5(10.6)	
**EBV viremia, n (%)**	19(27.1)	10(21.3)	0.519
**Administration of Rituximab before COVID-19, n (%)**	11(15.7)	4(8.5)	0.398
**Rituximab dosage, n (%)**			0.604
**⩾750 mg/m^2**	6(8.6)	3(6.4)	
**<750 mg/m^2**	5(7.1)	1(2.1)	
**Administration of immunosuppressive agents before COVID-19**			

CNI, n (%)	29(41.4)	10(21.3)	0.028
TKI, n (%)	5(7.1)	1(2.1)	0.399

Abbreviations: AML, acute myeloid leukemia; MDS, myelodysplastic syndrome; ALL, acute lymphoblastic leukemia; GS, granulocytic sarcoma; MM, multiple myeloma; CMML, chronic myelomonocytic leukemia; HCT-CI, HCT comorbidity index; GvHD, graft-versus-host disease; MAC, Myeloablative conditioning; RIC, Reduced-intensity conditioning; URD, unrelated donor; MSD, matched sibling donor; HID, haploidentical donor; ATG, Anti-thymocyte globulin; CNI, Calcineurin Inhibitor; TKI, Tyrosine kinase inhibitor; CMV, cytomegalovirus; EBV, Epstein-Barr virus; PB, peripheral blood; UCD, umbilical cord blood.

Supplemental Table 3: The comparison of mild/moderate and sever/critical infections

	Mild/Moderate (n=56)	Severe /Critical (n=14)	*P* value
**Sex（%）**			1
Male	26(46.4)	7(50)	
Female	30(53.6)	7(50)	
**Age, years (range)**	50(16-74)	54(18-69)	0.406
**Diagnosis, n (%)**			0.581
AML	19(33.9)	6(42.9)	
ALL	8(14.3)	3(21.4)	
MDS	4(7.1)	0	
GS	2(3.6)	0	
MM	20(35.7)	3(21.4)	
lymphoma	3(5.4)	2(14.3)	
**HCT-CI score, n (%)**			0.035
0	42(75)	7(50)	
1-2	13(23.2)	5(35.7)	
3-4	0	2(14.3)	
≥5	1(1.8)	0	
**Donor type, n (%)**			0.586
HID	27(48.2)	8(57.1)	
MSD	6(10.7)	0	
URD	2(3.6)	1(7.1)	
autologous	21(37.5)	5(35.7)	
**Graft type, n (%)**			1
Peripheral blood	53(94.6)	14(100)	

Peripheral blood and Umbilical cord blood	3(5.4)	0	
**Conditioning regimens, n (%)**			
MAC	33(58.9)	8(57.1)	1
RIC	23(41.1)	6(42.9)	
**ATG dosage, n (%)**			0.143
≥5mg/kg	23(41.1)	9(64.3)	
0-5mg/kg	33(58.9)	5(35.7)	
**Vaccination, n (%)**			0.519
0	13(23.2)	3(21.4)	
1 dose	2(3.6)	2(14.3)	
2 doses	25(44.6)	6(42.9)	
3 doses	16(28.6)	3(21.4)	
**Acute GvHD grade II–IV before COVID-19, n (%)**			1
No	49(87.5)	13(92.9)	
Yes	7(12.5)	1(7.1)	
**Chronic GvHD before COVID-19, n (%)**			0.577
No	52(92.9)	14(100)	
Yes	4(7.1)	0	
**CMV viremia, n (%)**	20(35.7)	7(50)	0.368
**CMV disease, n (%)**	1(1.8)	3(21.4)	0.023
**EBV viremia, n (%)**	11(19.6)	8(57.1)	0.015
**Rituximab for EBV viremia**	4(7.1)	7(50)	0.001
**Rituximab dosage, n (%)**			0.242
**⩾750 mg/m^2**	1(1.8)	5(35.7)	
**＜750 mg/m^2**	3(5.4)	2(14.3)	
**Administration of immunosuppressive agents before COVID-19**			
CNI, n (%)	21(37.5)	8(57.1)	0.23
TKI, n (%)	5(8.9)	0	0.575

Abbreviations: AML, acute myeloid leukemia; MDS, myelodysplastic syndrome; ALL, acute lymphoblastic leukemia; GS, granulocytic sarcoma; MM, multiple myeloma; CMML, chronic myelomonocytic leukemia; HCT-CI, HCT comorbidity index; GvHD, graft-versus-host disease; MAC, Myeloablative conditioning; RIC, Reduced-intensity conditioning; URD, unrelated donor; MSD, matched sibling donor; HID, haploidentical donor; ATG, Anti-thymocyte globulin; CNI, Calcineurin Inhibitor; TKI, Tyrosine kinase inhibitor; CMV, cytomegalovirus; EBV, Epstein-Barr virus.

Supplemental Figures

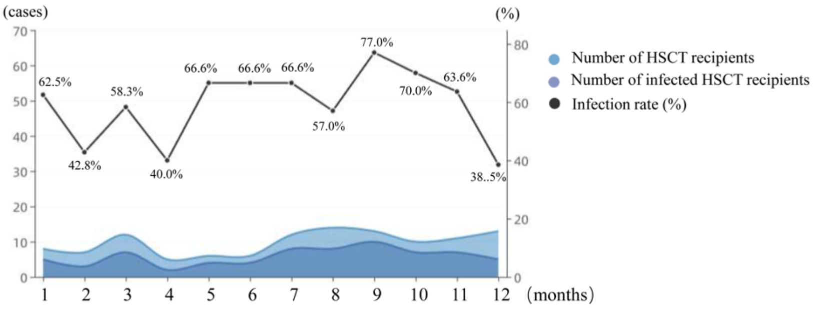

Supplemental Fig.1 Number of HSCT cases and percentage of SARS-CoV-2 infection according to the date of exposure.

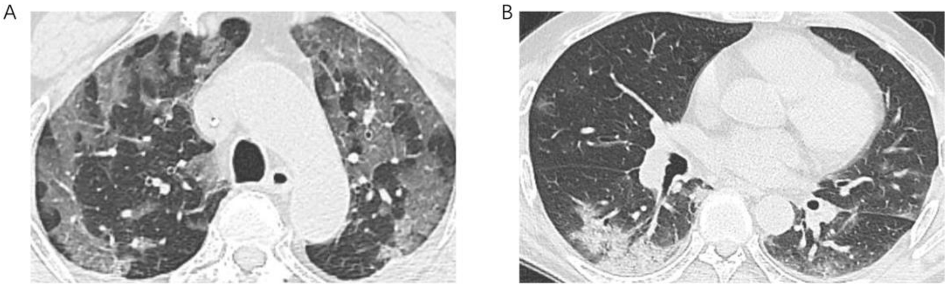

Supplemental Fig.2 CT findings of COVID-19 pneumonia (lung window). (A) extensive areas of ground-glass opacity involving bilateral lungs. (B) consolidation with subpleural distribution

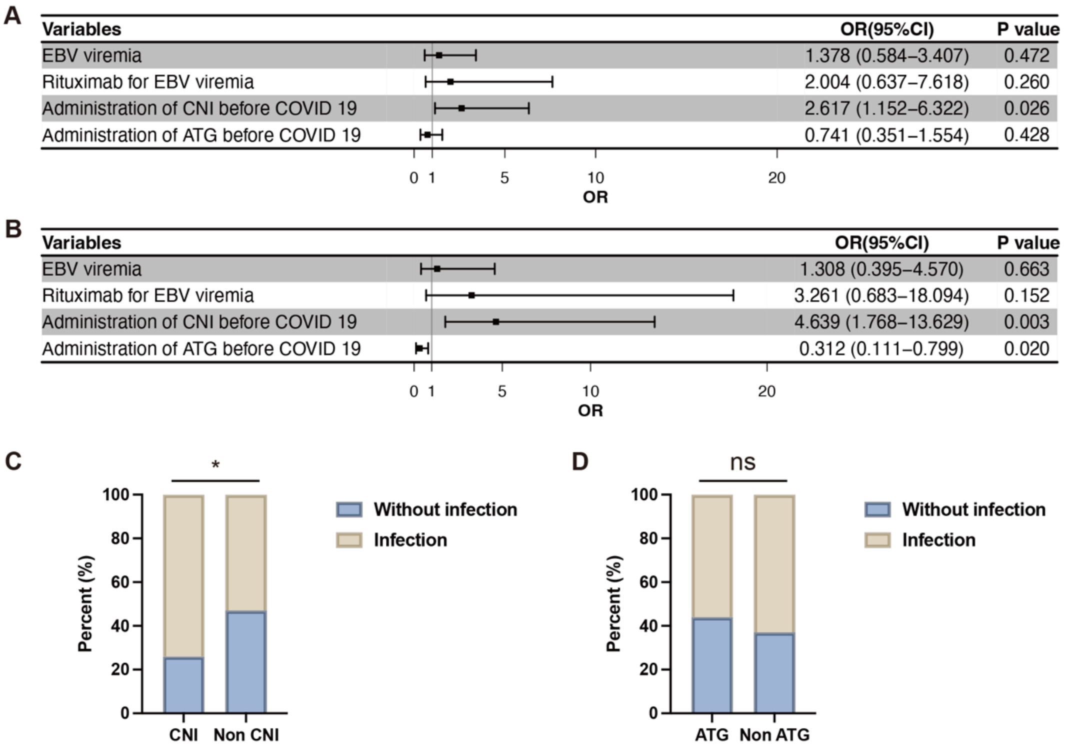

Supplemental Fig.3 Factors affecting COVID-19 infection. Univariate (A) and multivariate (B) analysis of predictors at diagnosis of COVID-19. Comparison of COVID-19 infection rate between patients with or without CNI (C) or ATG (D) administration. OR, odds ratio. CI, Confidence Interval. *p<0.05.
